# Power imbalances and equity in the day-to-day functioning of a north plus multi-south higher education institutions partnership: a case study

**DOI:** 10.1186/s12939-024-02139-x

**Published:** 2024-03-15

**Authors:** Silondile Luthuli, Marguerite Daniel, J. Hope Corbin

**Affiliations:** 1 Department of Health Promotion and Development, University of Bergen, Bergen, Norway; 2 Centre for Rural Health, University of KwaZulu-Natal, Durban, South Africa; 3 Department of Health and Community Studies, Western Washington University, Bellingham, WA USA

**Keywords:** Partnerships, North-south, Higher education institutions, Health research, Power imbalances, Equity

## Abstract

**Background:**

Partnerships between Higher Education Institutions (HEIs) in the global north and south have commonly been used as a vehicle to drive global health research and initiatives. Among these initiatives, include health system strengthening, research capacity building, and human resource training in developing countries. However, the partnership functioning of many global north-south partnerships still carry legacies of colonialism through unrecognized behavior patterns, attitudes, and belief systems in how they function. Even with research literature calling for a shift from equality to equity in the functioning of these partnerships, many still struggle with issues of complex and unspoken power dynamics. To understand the successes and challenges of north-south partnerships, this paper explored partnership development and functioning of a northern and multi-southern HEIs partnership focused on nutrition education and research.

**Methods:**

A qualitative research approach was used; data were collected through in-depth interviews (IDIs) with questions developed from the Bergen Model of Collective Functioning (BMCF). Thirteen IDIs were conducted with partners from all institutions including stakeholders.

**Findings:**

The partnership was built on the foundation of experiences and lessons of a previous partnership. Partners used these experiences and lessons to devise strategies to improve partnership inputs, communication, leadership, roles and structures, and maintenance and communication tasks. However, these strategies had an impact on partnership functioning giving rise to issues of inequitable power dynamics. The northern partner had two roles: one as an equal partner and another as distributor of project funds; this caused a conflict in roles for this partner. The partners distinguished themselves according to partner resources – two partners were named implementing partners and two named supportive partners. Roles and partner resources were the greatest contributors to power imbalances and caused delays in project activities.

**Conclusion:**

Using the BMCF to examine partnership dynamics illuminated that power imbalances caused a hierarchical stance in the partnership with northern partners having overall control and power of decision-making in the partnership. This could impact the effectiveness and sustainability of project in the southern institutions going forward.

## Background

Partnerships between countries in the global north and global south have been recognized as crucial and beneficial to southern countries in addressing health-related challenges and promoting global health for all [1–3]. Countries in the global south often face health-related challenges such as high rates of infectious diseases, non-communicable diseases, inadequate healthcare infrastructure, and limited access to healthcare services [4–7], leading to poor health indicators and outcomes. Many of these health challenges can be argued to be products of systematic deprivation through colonialism and unfavorable economic policies and programs at the country level [8].

Partnerships with governments and health and education institutions in High-Income Countries (HICs) have assisted low-middle-income countries (LMICs) in combating health-related challenges and developing relevant interventions and policies for health [9–12]. Moreover, partnerships between Higher Education Institutions (HEIs) in the global north and global south have commonly been used as a vehicle to drive global health initiatives and research between HICs and LMICs. These partnerships are often focused on health systems strengthening, research capacity building, and human resource training to improve health through human resources [9, 13–18]. However, Khan et al. [19] and Whitehead et al. [20] argue that many of the north-south partnerships still carry legacies of colonialism through unrecognized behavior patterns, attitudes, and belief systems that are adopted by these partnerships.

The success of north-south partnerships between HEIs for Global health and health research in achieving their outcomes has been documented [21–24] but literature reports on the operation and functioning of these partnerships are scarce [25]. Key characteristics of the success of global health partnerships include a common understanding of vision and mission, mutual respect and benefits, trust, good communication, and clear partner role distribution and expectations [21, 26]. Even with these key characteristics in place, many global health partnerships still face challenges with power dynamics and these are often rooted in colonial legacies that perpetuate the paternalistic approach of HICs on LMICs [1, 27–29].

Crane [1] and Geissler [27] further mention that the conceptualization of global health exacerbates power dynamics because it often pairs countries that are unequal to improve or promote health. Over the years there has been a shift toward equity in global health research between HICs and LMICs [30–33], with researchers recognizing that inequalities do exist in global health partnerships but strategies need to be implemented to mitigate power dynamics. Key areas of improvement to flatten power dynamics include recognizing ethical issues within partnership functioning [32], research should focus on local health priorities [34–36], acknowledge capacities and limitations to contribution in partnership [32], recognize different skill sets, training background, resources, and funding [32, 37], recognize local expertise [38], build trust between funders and southern partners [32, 36], and transparent communication from beginning of partnership [39]. Even with these key strategies to flatten power dynamics in partnerships, and characteristics of successful partnerships mentioned above, many global health partnerships between HICs and LMICs still experience challenging power dynamics, therefore, more research is needed to understand the functioning of north-south partnerships between HEIs, perhaps from the standpoint of day-to-day operation of these partnerships.

Having identified issues raised in the literature, a case study using categories from the Bergen Model of Collaborative Functioning (BMCF) will be helpful to trace the pathway to understand partnership successes and negative processes that impact partnership functioning. The BMCF framework has been used in some global health partnerships with power dynamics rising as key issues [39–41]. However, literature that focuses on the day-to-day functioning of these partnerships is scarce. This paper describes a study investigating the partnership development and functioning of a northern and multi-southern partnership focused on nutrition education and research by exploring how different partners understood and contributed to: (1) mission, partner resources, and financial resources (2) Leadership and roles and structures (3) input interaction and communication (4) production and maintenance tasks.

### The case

PROJECT-2 is a North-multi-South partnership between Higher Education Institutions (HEIs) focused on nutrition education, research, and capacity building. Four institutions are collaborating on PROJECT-2. The partners comprise ‘supporting’ partners – Northern partner (N1)^1^ and one Southern partner (S1) – and ‘implementing’ partners – the remaining Southern partners (S2 & S3). The terms ‘supporting’ and ‘implementing’ were informally developed by the partners based on how they perceived their roles in the partnership. PROJECT-2 is an extension of a former project, PROJECT-1, with a new partner (S3) joining the partnership. The project is funded by a Northern government funding agency referred to as FUNDER in the paper. The aim of FUNDER agency is to support projects that promote global development, green living, and ending world poverty. Much like the project, the funding agency has funding programs – FUNDING-1 refers to funding for previous partnership, and FUNDING-2 funding for the new project, PROJECT-2. The FUNDING program aims to strengthen the capacity of higher education institution in developing countries to produce higher-quality graduates, higher-quality research, and inclusive higher education. According to FUNDING, the projects must be based on partner institutions’ identified needs and contextual needs.

PROJECT-2 is built upon learnings and experiences from PROJECT-1. The main aim of the partnership is to address the shortage of research capacity to inform the development of locally relevant evidence-based policies in two low-income countries (where S2 and S3 are based), using nutrition as the vehicle for capacity building. The objectives of the partnership are (1) to develop and implement a master’s and PhD Nutrition program (2) to establish research capacity building (3) to inform the development of locally relevant nutrition policies. The countries S2 and S3 were chosen as research sites because they were among countries on the FUNDING-2 list as potential collaborating countries according to FUNDER country’s development policy and they presented poor nutrition health indicators. At the core of PROJECT-2 was to build a partnership that was mutually beneficial to all partners and founded upon the health and nutrition needs of S2 and S3 at the forefront of the partnership.

### Conceptual framework

**Fig. 1:**
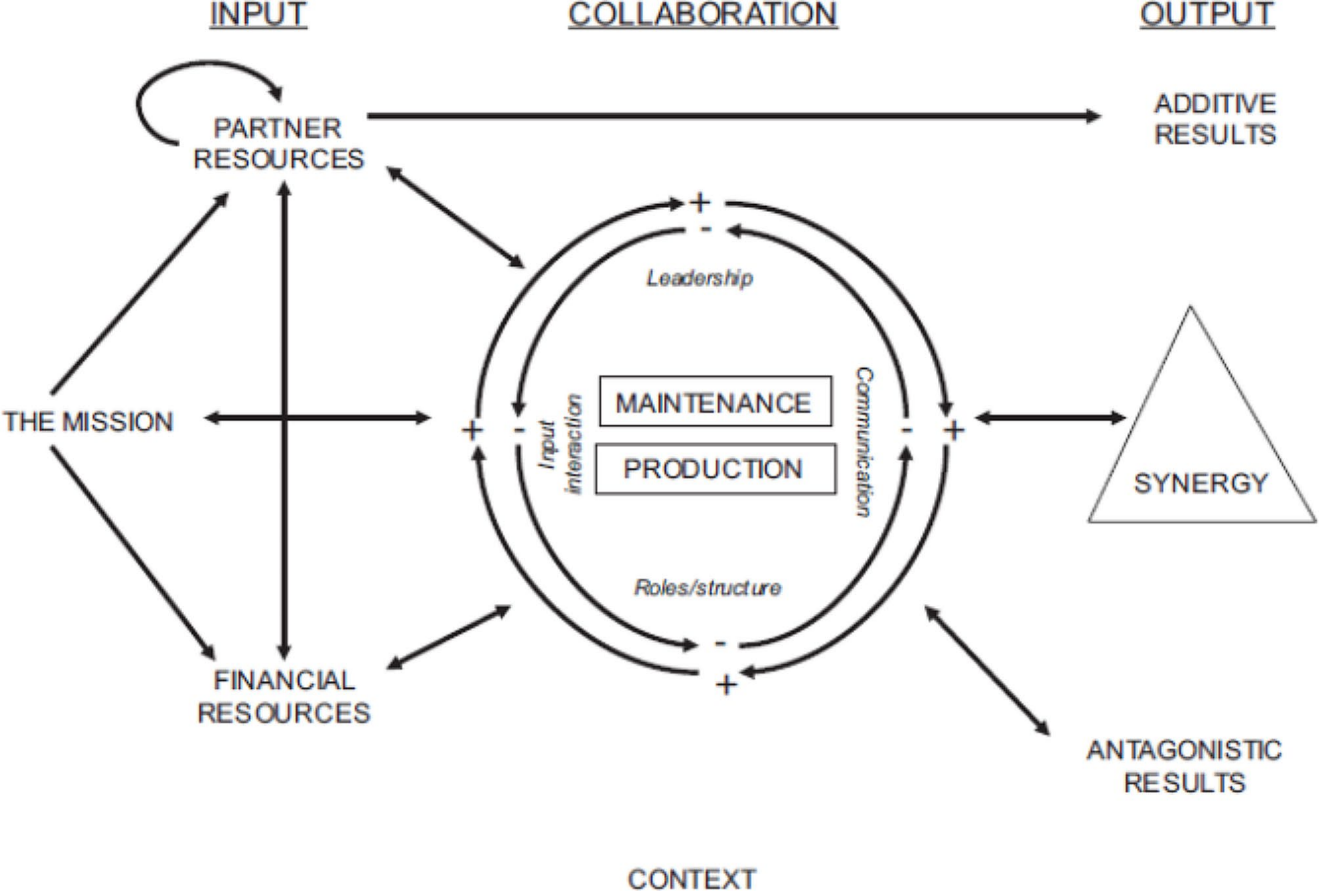
The bergen model of collaborative functioning [44]

The study used the Bergen Model of Collaborative Functioning (BMCF). Most details about the model were drawn from Corbin and Mittlemark [42] and used as a reference in this section. The systems model provides an input-throughput-output analytical frame to examine partnerships. The inputs and throughputs interact and function together to produce outputs that feedback to the partnership positively or negatively, all of this happens within a context (Fig. 1). The inputs to the partnership are the mission, partner resources, and financial resources. The mission is the vision and objectives of the partnership in how the project will function. Partner resources refer to the skills, knowledge, commitment, connections, and other attributes that humans contribute to the partnership. Financial resources are all the monetary and material investments in the partnership.

The throughput is the collaborative context, the inputs enter the context and interact positively or negatively with elements in the collaborative context as they work on the production tasks (related to the mission) and the maintenance tasks (related to administrative duties). There are four elements within the collaborative context – input interaction, leadership, roles/structure, and communication. These elements create dynamics and reinforce cycles within the collaborative context through their interactions.

The outputs of the collaborative context may be additive, synergy or antagony. Synergy is the intended product of partnership, all the partners bring resources, skills, etc. to bring forth a product bigger than their individual effort. Antagony is not only the failure to reach synergy in the partnership but also the wasting of partner and financial resources so that more is consumed in the partnership process than produced in the partnership. Antagony is any tension in the interaction between collaborative partners that causes interferences, tension, and counter-productivity [43].

The model was appropriate for the current study because it allowed the researchers to explore various aspects and characteristics of the PROJECT-2 partnership. The model has also been successfully used in many health research partnerships with countries in the global north and south [39–41, 43, 44]. In this paper, only the input and throughput sections of the model were used during the analysis of the data.

## Methods

A qualitative research approach using in-depth interviews (IDIs) was used in the study to explore the partners’ understanding of the mission and the functioning of the PROJECT-2 partnership. Interviews were conducted with partners and stakeholders involved in the project.

### Participants and recruitment

The study population included participants from all the partner institutions and stakeholders from the relevant Government Departments in the research site countries. Participants included Principal Investigators (PIs), administrators, professors, researchers, PhD candidates, and representatives at country level in research site. A purposive sampling strategy was employed in which participants were selected based on their expertise on the subject matter [45]. Using purposive sampling enabled the researcher to select participants who had extensive knowledge and experience of the project – this was determined by the participants’ involvement in the following project activities: (1) involvement in proposal development (2) engaged in daily activities of the project at individual institution (3) Attending monthly and annual partner meetings, and (4) administrative duties of the project. Recruitment began with the first author attending all the partner virtual monthly meetings to take note of key members from each institution and their involvement in the project. The first author was part of the list of people attending project activities, this included the first annual in-person workshop with the partners. In the annual meeting, the first author introduced the study and invited all participants to participate. Partner representatives who did not attend any monthly meetings or the physical annual meetings were excluded from the study.

### Data collection

Semi-structured IDIs were conducted with participants over a period of five months, between February and June 2022. A total of 13 interviews were conducted by the first author. These were three IDIs with members from N1 institution, three IDIs with members from S1, three IDIs with members from S2 and three IDIs with members from S3, and one stakeholder representing country level department in S3. One partner representative was unavailable for an interview and was therefore excluded from the study. An interview guide was developed using the BMCF model to structure topics to be included in the interviews. Topics included in the guide were the partners’ understanding of the mission, contribution of each institution to the partnership, project funding, distribution of roles, views on project leadership, and expected outcomes from the project. Seven interviews were conducted in-person, of these five were done during the partner’s annual meeting. Five interviews were conducted virtually using ZOOM, and one participant requested to provide written answers to interview questions due to the language barrier. All interviews were done in English language.

### Data analysis

All interviews were either audio or digitally recorded, using audio recording device for in-person interviews and digital recording through Zoom during virtual interviews. The interviews were 13 to 64 min long. Interviews were transcribed verbatim using a transcribing software Amberscript and Zoom transcribing. All interviews were quality checked by the first author to ensure that everything was accurately captured during transcribing process. The first author listened to audios and read transcripts and made necessary corrections. Analysis was conducted by the first and second authors. The steps of thematic network analysis were followed to analyze the data [46] and NVIVO v12 was used to manage the data. A hybrid approach, using both inductive and deductive approaches, was employed to guide the analysis process. The inductive approach was used in the initial stages of coding and analysis, followed by deductive approach during the process of developing organizing and global themes (see Table 1). The authors (SL, MD) read all the transcripts to familiarize themselves with the data and met frequently virtually and physically to discuss coding process, develop codebook, and themes coming from the data. The final global themes that emerged are structured according to the BMCF model: context, input, and throughput.

**Table 1:** coding framework

Basic themes	Organising themes	Global themes
*Building on PROJECT-1 *Expanding structure	Partnership background	Context
*Understanding the mission of the project *Vision of the project *Institution gains from the project *Expected outputs *Personal learning and goals	Mission	Inputs
*Implementing partners *Supporting partners	Partner resources	
*Funders and funding	Financial resources	
*Role in fulfilling the mission	Input interaction	The collaborative context (Throughput)
*Roles and responsibilities *Leadership *Leadership in the implementing institutions *Teaching and supervision tasks *Research tasks	Production tasks	
*Team meetings *Admin tasks *New administrative role in northern institution *Practical and contextual challenges in communication	Maintenance tasks	

### Ethical considerations

This study’s data management plan was approved by the [name of institution] for Research Data. Signed informed consent was obtained from all participants before each interview and all identifying information was removed to ensure confidentiality.

## Findings

The findings are presented according to the structure of BMCF. Elements of the model used are context, inputs, and throughputs which became the global themes of the findings with basic and organizing themes reflecting the initial experiences of participants in developing and establishing the partnership.

### Context

#### Partnership background

##### Building from PROJECT-1:

coming to the new partnership, the participants expressed that PROJECT-2 is founded on experiences and relationships, achievements, and challenges learned from PROJECT-1. This had an influence on changes that were planned for this second round of the project. Participants presented mixed views about their experiences of being involved in PROJECT-1. Some participants commented on the functioning of the first project and how that might impact the new partnership. Mentioning that “*what is good, is that most of the partners are the ones that were involved in PROJECT-1. So we already know each other and I know how we work, I know we had no problem like collaborating” (Participant 7, IP*^2^*).* Another participant mentioned the operation of the first project being poorly managed, commenting specifically that:

*a lot of things in PROJECT-1 were done on a fairly ad hoc basis… we had meetings here and there quite often, but people didn’t always turn up… It was… sort of normalized that if you have something else to do, then you would not go to the PROJECT meeting (Participant 8, SP).*

In some cases, the outcomes of the first project were perceived to have benefited one partner more than others in the whole project.

*… S2 received an extension budget to develop a e-Learning system. And we did just before Covid pandemic and the system helped the school to continue with delivering teaching and courses remotely (Participant 10, IP).*

PROJECT-1 achieved several other positive outcomes, for example “… *they managed to kind of get out with 40 out of the 41 students that got their masters in the partnership… there will be four PhD candidates also” (Participant 3, SP).* The participant also commented on the good relationships between the partners on which the second project was built. However, the participants also expressed that there were numerous challenges that affected the functioning of PROJECT-1. These included issues with security in Country S2 “*… where it was not possible to travel because of conflict” (Participant 3, SP)*, language barriers which had an impact on communication between partners and students, communication with the funders, and relationships with institutional boards.

##### Language as barrier to communication:

PROJECT-1 was operated in English, which meant all communication between partners, and students was done in English. However, there were misunderstandings between partners, students, and institutional boards about the requirements of the project and that of the institution for academic programs. As a requirement of the project, the students had to write and defend their theses in English to pass the program. However, S2 institution only accepted work done in French. “*We learnt that in PROJECT-1, we forced the students to write their thesis in English, and just yesterday we learnt that many of those theses were back translated into French and defended in French” (Participant 3, SP).* The participant went on to explain that had the country steering committee told them at that time, they could have come up with alternative solutions. Communication between funders and institution in Country S2 was another challenge expressed by one of the participants. The institution in Country S2 experienced issues with reports to funders that were embedded in language barrier between the partners.

“*… since our accountant, is not that strong in the English, it was a bit complicated and we had to write rewrites [of reports]… we had some troubles like spending some of the money that we requested” (Participant 7, IP).*

Going into PROJECT-2 partnership, there were **changes implemented by the funders and by partners**. The funders made the northern partners in charge of distribution of funds to the other partners and the coordination of the overall project.

*Before in PROJECT-1, they gave the responsibility to each south partner, but now they have given it to the N1 partner to coordinate everything that happens in the project when it comes to administration, like the money transfer and all of these things (Participant 1, SP).*

Some of the participants found the change implemented by the funders frustrating because they introduced uncertainties and delays with project activities “*… instead of us as project, me as project manager relating to FUNDER directly, we have to now go through the N1 Secretariat. And they have a quite unclear role… but we know we have to wait” (Participant 3, SP).*

Another change implemented in the project is the exchange of students from the northern partners to the southern partner institutions; whereas previously only southern partner students had opportunity of attending courses in northern institution. Participant 3, SP explained the introduction of a new practice:

“*… a set of N1 students will come and join students in Country S3 and Country S2 to see what learning opportunities are there from that process, it will be quite interesting”*.

##### Expanding structure:

the partnership brought changes in the form of a new partner joining the project. The participants mentioned that “*… bringing in [a] new partner has strengthened the program…” (Participant 3, SP).* The participant went on to say “*… the new partner has shown to be, I think, very strong and fitted extremely well into the program and taking responsibility and has been a very positive addition to the program”.* Bringing in a new partner has also brought a sense of commitment from the old partners.

“*And as I see the old participants who have been working, they are committed. Each site wants to make sure that they attain their goals” (Participant 4, IP).*

Involving stakeholders in the partnership activities was an important aspect of expanding the structure and developing context-relevant research agenda, focused on the health priorities of the southern partners. The partners were in communication with representatives from various departments at country level to get support and a list of research priorities in the research site countries.

The stakeholder in one of the sites went on to say that they will be working closely with the institution as research hub to provide access to data that students can use in their studies.

*I have research ongoing, I have platforms that can give access to data via other government sources and… they can access the information they need for their research projects (Participant 12, IP).*

Going into PROJECT-2, the context (background of PROJECT-1) became the foundation of the collaborative context for PROJECT-2. The partners made changes and developed strategies to mitigate the challenges that were previously experienced. The program was set to continue to operate in English with planned strategies to enhance communication and engagements between students and partners.

### Inputs

Inputs emerged as a global theme that was pivotal in the development and functioning of PROJECT-2 partnership.

#### Mission

##### Understanding the mission of the project:

the participants had different understandings about the mission of the project. For the majority of the participants, the mission of the project was related to developing human resources and building strong collaborations between countries. As highlighted by one participant who mentioned that PROJECT-2 mission is similar to PROJECT-1 mission, and stated that the mission is.

*… to establish a strong collaboration for improving nutritional epidemiology, research and education in nutritional epidemiology in Country S2 and Country S3… so improving nutritional epidemiology research and education in both countries (Participant 10, IP).*

For another participant, the mission of the project was to establish a master’s and PhD program in nutrition research in Country S2 and Country S3.

*And I think, looking back to the kind of call from the donors, it’s that building, that the higher education, which is the main objective of, of FUNDING. And by that I think we have succeeded in PROJECT-1, and the hope that we can succeed in PROJECT-2 in kind of building this master, and PhD capacity (Participant 3, SP).*

The participant continued, highlighting the importance of research, “*where the big challenges is on the research,… because you can’t have research-based master’s program and PhD programs without having a good research project”.*

In contrast, some participants had uncertainties about what is the mission of the project. Participant 8 (SP), mentioned she could not remember what the mission was about “*… participatory research and education to develop to develop skills, something like that was what we wrote… but I think it’s empowering”.* Whereas, participant 5 (IP), was convinced that the project “*… does not have the mission yet. We have objectives… to address the human resource shortages… creating evidence that can also support the challenges of nutrition related conditions”.*

##### Vision of the project:

the participants also expressed different views about the vision of the project. One of the participants explained the vision of the project is working together towards a common goal and working well together based on relationships built in the previous project.

*… we all want the project to do well, and that we all do have a fairly common vision into as to where we’re going and that we’ve worked together for all this time, not Country S3, but the other people without any conflict, really (Participant 8, SP).*

Another participant, expressed that like the mission, *the vision is not yet set (Participant 5, IP).*

##### Institution gains from the project:

when asked about the gains of each institution in the project, the participants found it easy to articulate the gains of the two southern partners where the project will be implemented but difficult to describe the institution gains of the northern partner and the other southern partner. By the end of the project the partners in institution S2 and institution S3 would have gained master’s and PhD graduates in the field of nutrition, an opportunity for staff and students to develop careers and broaden horizons, facilitation and teaching skills, distance learning skills and materials, and sound research. The stakeholder in one institution made it clear that the government department at the country level will also benefit from the project in different ways.

*… my gains are two-fold… I do have a lot of data, some are redundant, that are sitting here, then that would benefit a lot from having somebody manipulate, model, and give us more information on it, so that’s one. I will have hands on local information on what is happening on the ground in terms of nutrition (Participant 12, IP).*

The participant went on to say that once the project starts producing graduates there will be “… *a bigger pool of employees who are competent in manipulating and analyzing and even collecting data but more importantly, conceptualizing the design of different research projects”.*

For partners in Country N1 and Country S1, the institution gains were unclear. One participant mentioned that there is nothing that the N1 partners are expecting to gain in the project, he explained that working in north-south partnerships “… *is our mission. So, if we are able to complete our mission to train staff members in lower-middle-income countries, we are just happy” (Participant 2, SP).* The participant expanded to say that the institution gains of the Country S1 partner “*… will be expanding the horizons of the Centre, knowing more about Africa outside Country S1”.*

##### Expected outputs:

the participants were asked about their expectations in working on the project to understand the outputs. Much like institution gains, some partners were expecting to get graduates and researchers in nutrition research by the end of the project. Other outputs mentioned by the participants are divided into short-term and long-term outputs. For short-term outputs, participants mentioned getting started with the program in Country S3, the partners being focused, structured, and output orientated, large research studies for students, and getting students’ research proposals in good English and through N1 ethics processes. The students in S2 and S3 institutions had to submit their research proposals for ethical approval to northern institution as part of their PROJECT-2 requirement.

The participants in institution S2 also mentioned that there were changes in the curriculum due to a new teaching strategy that was implemented by the institution. As an outcome, the participants, are anticipating a smooth transition that will honor expectations from both project partners and institution. Long-term, the participants mentioned that they expect to see research publications coming from the project, more partnerships and other funding opportunities, and improvement in nutrition and food indicators in both research site countries.

##### Personal learning and goals:

the participants were asked what they would hope to have personally gained by the end of the project. These included improving language communication, new skills in financial management and administration, writing skills, communication skills, use of technology for blended learning, online teaching and assessment skills, development of online/distance learning courses skills, research skills, teaching skills, staff development, and opportunities to exploring other cultures. A common skill mentioned by the partners was the management skill in relation to leading organisations and multinational projects.

#### Partner resources

The participants distinguished themselves according to **implementing partners** and **supporting partners** in the project. These labels are according to the role distribution and resources that each institution is bringing into the partnership. The supporting partners, Country N1, they are *“… the one who like funds the project… they also have this rich contribution on the management of the whole project” (Participant 10, IP)* and also provide support in supervision, teaching, and research. The institution in Country S1 team brings skills related to training and research to the partnership.

*I think the Country S1 team brings with it, strength in the development of training materials and development of research proposals, development of tools, and those kind of aspects within the research (Participant 11, SP).*

With the implementing partners, the resources they bring to the partnership were related to skills in teaching and curriculum development of the program. “*I understand that the [role] of S3 and S2 will be more into the teaching of the curriculum that we have developed” (Participant 6, IP).*

#### Financial resources

##### Funders and funding:

with the changes introduced by the funders in PROJECT-2, this presented mixed views from the participants. For the northern partners, this was an added administrative duty that caused frustration and delays in the progress of project activities for all the institutions.

*… because FUNDER… have changed the way they organize the structure… there was more than half a year delay because of the contractor issues… and think it will maybe take even maybe half a year or a year to have a full circle in (Participant 1, SP).*

### The collaborative context (throughput)

#### Input interaction

In the study, this was understood as the participants’ understanding of their **own personal contribution and the different institutions’ contribution in fulfilling the mission**. Many of the participants made a link between the roles they play in the partnership as a key contribution they are making in fulfilling the mission of partnership. One participant felt his role in the partnership was not clear and was not sure how he will individually contribute to fulfil the partnership mission.

When describing the contributions of each partner institution to the mission of the partnership, the participants had clear understandings of what the supporting partners were contributing. For example, this particular participant explained that N1 institution’s contribution to the partnership was *“… to improve the opportunities for training and pushing the knowledge agenda forward in Country S3 and… Country S2” (Participant 2, SP).* Other participants had a clear understanding that S1 was contributing through providing research skills, training, teaching, and supervision; as highlighted in the resources sub-theme.

The participants were not so clear in describing the role of the implementing institutions in fulfilling the partnership mission. The implementing partners themselves and some other partners kept referring to the roles of teaching and supervision as key contributions to the fulfilment of mission.

The supporting partners often had comparative and competitive descriptions of the individual contributions of the implementing institutions, as seen in the leadership theme. Participant two in particular, felt that one specific partner was not contributing much resources into the partnership but needed the most support to fulfil mission in institution, see below.

*… with Country S2, I think it will be like the small brother in the group, who is contributing the least, and needs the most guidance, and the local issues I talked about earlier is, adding to this… (Participant 2, SP).*

#### Production and maintenance tasks

Even though the main focus of the paper/data collection was to understand the initial stages of partnership development and implementation, however, the partners did have plans and activities for production and maintenance tasks. In this section, we present findings of how different activities had a positive and negative interaction with each other during the early stages of partnership development and implementation.

#### Production tasks

Production tasks include activities that are undertaken for the purpose of achieving the mission in a partnership. Two characteristics (roles and leadership) are important in understanding how production tasks are conducted to produce the intended outcomes in the partnership.

##### Roles and responsibilities:

All the partners were aware of the various roles that they individually play in the partnership and there was an awareness of the various roles and responsibilities that institutions play in fulfilling the mission of the project. One of the partners highlighted that there was a fair distribution of responsibilities with regard to project activities, this helped *“… everyone know what is expected of them” (Participant 7, IP).* The roles mentioned by participants include administrative and financial management roles, project manager/coordinator, principal investigators in each site responsible for overseeing the implementation of objectives and research activities in institutions, being a teacher and co-supervisor in the project, coordinating the development of curriculum in the institution, being a stakeholder and research hub, and being PhD candidates in the project. One of the participants expressed that coming from a northern partner, their role was conflicting because the partnership is structured to be equal “*… where we are on the very egalitarian basis, but in the same time, the N1 plays a role as a controller of the others, because we are the ones that are report to the donors” (Participant 3, SP)*, they felt that they have more power to control roles and responsibilities in the partnership.

##### Leadership:

the partnership has leaders in each institution but there is also an overall leader/manager in the project, the PI from Country N1. The project manager’s role in the partnership is to organize partner meetings (virtual and physical), facilitate the meetings, take notes during meetings, and liaise with the funders. When asked about leadership style in the partnership, some of the partners expressed that leadership is good, encourages shared decision-making among partners, there is openness to share the leadership role, and the project manager does “*… not try to control the way things are going and gives everybody the floor and let everybody speak…” (Participant 8, SP)*, and this was perceived as a good model of leadership and an improved leadership style from the previous, PROJECT-1 partnership, leaning towards a more collaborative orientated project.

However, some of the N1 partners were concerned about the power dynamics in the partnership. They felt the northern side of the partnership was *“… imposing a lot of things on the partners…” (Participant 1, SP)* because of their dual role and multiple responsibilities in the partnership. The northern partners felt that some of these responsibilities needed to be shared among partners in order to flatten out the power dynamics. One of the participants explained that a platform to share some of the project administrative duties with the project manager was opened in the first partner meeting but none of the other partners took up the offer.

When describing the **leadership in the implementing institutions**, the partners often compared the leadership styles of these two leaders in implementing institutions. The participants spoke of one leader as driven, clearly understanding the mission, committed, wanting to build a future and career, and bringing wealth of knowledge and experience to the partnership. Whereas, when speaking about leadership in the other institution, some participants alluded to underlying issues in leadership that affect the functioning of project in that institution.

##### Teaching and supervision tasks:

one of the main production tasks in PROJECT-2 was the development and implementation of the nutrition research program (curriculum) in S3 institution and continued support at S2 institution. Other teaching and supervision tasks in the partnership included finding strategies for blended learning in the implementation institutions.

*In PROJECT-1 we had made the recommendation that we move to mixed methods teaching platform, where one would not teach only face-to-face… but rather use multiple methodologies for teaching and I think that from the workshop its very clear that that’s the way they want to go (Participant 11, SP).*

##### Research tasks:

building a research agenda that addresses nutrition priorities and policy at country level was important for the project. Research studies that would be conducted by the students in the project had to link to country priorities, and this was done through working in collaboration with government departments in each implementing country and they provided a list of research priorities in nutrition for the country. Ensuring that research studies conducted in the project address policy change and the need for evidence for interventions or publication in the implementing countries was important for the partners. As highlighted by participant 5 who explained that project would be.

*… generating evidence that can address the dearth of evidence, in these countries for policymaking processes to address the nutrition challenges, and together we can also address issues with regard to health and welfare of the society (Participant 5, IP).*

However, in developing plans and strategies about the research agenda, the partners had agreed that having “*… bigger research projects that involve both master’s students and PhD students around a few projects… instead of… very small studies” (Participant 3, SP)* would work best for the project. This would allow the partners and project at large to get an in-depth understanding of the topic under investigation.

#### Maintenance tasks

Maintenance tasks are activities that keep the partnership functioning, these include administration duties, meetings, grant writing, and writing reports. Communication is an important characteristic of maintenance tasks. Maintenance tasks do not affect the mission of the project directly but play a significant supportive role in its achievement.

##### Team meetings:

the partners had regular virtual meetings to discuss project progress in each site and updates on project activities. As highlighted by Participant 4, who mentioned that the meetings were important in getting an understanding of activities they have to do as an institution and “… *reporting what we have been doing and what has been done and what needs to be done”.* The partners had their first in-person meeting/workshop and this provided partners an opportunity to engage with one another better. The workshop also provided partners “… *clarity in terms of what are the expectations from the project” (Participant 5, IP).* During discussions in the workshop, a decision was made that principal investigators (PIs) from all the institutions should have their own meetings to discuss “*… issues that need to be really interrogated that not everybody is agreeing with or if there is issues where one partner is lagging a bit behind…” (Participant 11, SP).*

##### Admin tasks:

the partners had to prepare budgets and reports for the funders about the first period of the project. One of the partners had delayed submitting the budget because they were unaware of the procedures. This particular partner explained that being part of the monthly meetings assisted in getting clarity about what is expected of them during reporting.

*I had some delays in submitting reports… but from being a member of those meetings, then I was becoming aware that I was supposed to do this and this… there is a budget, but we were required to prepare some six-month budgets for supporting some of the activities that are being done (Participant 4, SP).*

The same participant continued to express dissatisfaction about how administrators in the partnership have limited chance to interact amongst each other and learn from one another. The participant suggested that administrators should have their own workshops or maybe zoom meetings where they can learn from each other.

##### New administrative role in northern institution:

the overall administrative role and management of the project were operated by the northern partner and this caused frustration in the N1 partners because there was a lack of clarity of what is expected of them from the funders and there was concern that the role adds another dimension in the power dynamics. There was also concern that the shift in financial management duties may limit opportunities for capacity building for the south partners. Participant 3 (SP) explained that FUNDING-1 experienced numerous admin challenges in the previous project which led to moving all project management duties to northern partners. However, the shift in administrative duties was welcomed with gladness for one of the south partners because this meant they do not have to interact directly with the funders. This made their work easy as they were often unfamiliar with funder’s procedures of reporting.

*We were like directly responsible over all the things are related to financing, with FUNDER. But now we have to pass through the N1 [institution] which is a very good like way of doing things because actually, they are more accustomed to working with FUNDER agency and it makes things very easy for us (Participant 7, IP).*

##### Practical and contextual challenges in communication:

in the first year of PROJECT-2, the partners communicated mostly virtually through emails and Zoom meetings due to travel restrictions caused by COVID-19 pandemic. During that time, the partners were writing a funding proposal virtually and a lot of challenges related to communication were experience; some of these issues transferred to physical communication when the partners eventually met. There were concerns about misunderstandings when everything was done via text or zoom in a partnership and also there was often confusion on who was to be invited into zoom meetings. Some partners felt that communication was clear and structured with everyone knowing what is expected of them in the partnership. Other partners expressed a view that more physical meetings would strengthen relationships in the partnership and perhaps smaller groups within the partnership would be beneficial for better communication and shared experiences among different roles in the partnership. Participant 1 (SP) highlighted that perhaps it would be better “*… to arrange in these workshops to get more in smaller groups that is maybe easier to talk and to communicate and share experiences when you’re in that small group”* and separate these groups according to different roles in the partnership.

Culture and language were also an added layer of dynamics in the partnership that had an impact on communication. The partners come from diverse cultural backgrounds and speak different languages, an incident that happened during the first partner meeting brought awareness to the partners on the need to be considerate and respectful of different cultural contexts in the partnership. In this incident, one partner spoke harshly to another partner during a team meeting sending waves of shock among partners.

*I think it… speaks to a lack of understanding of culture and norms. And I think that working in a diverse cultural background, diversity of cultural backgrounds, we need to be mindful (Participant 11, SP).*

## Discussion

Using the Bergen Model of Collaborative Functioning (BMCF), this paper explored partnership development and functioning of PROJECT-2 as well as partners’ understanding and experiences of partnership. In this study, we found that PROJECT-2 was built on an existing partnership among the majority of the participants plus one new partner. Many of the participants alluded to the previous partnership experiences, PROJECT-1, having a significant impact on the establishment and overall functioning of the current partnership. These experiences were both positive and negative during PROJECT-1. Negative experiences in PROJECT-1 included poor management of the project, skewed benefit ratio between the partners, and poor communication between partners, funders, and institutions. These became the backbone of key changes intended in PROJECT-2, including the expansion of the nutrition program by introducing a new partner and involving stakeholders to influence the development of a context-relevant research agenda. The positive experiences included good working relationships, successfully developing and implementing master’s and PhD programs, and producing graduates from the program.

As a way to increase the chances of success in north-south partnerships, many authors have suggested that partnerships should be anchored on a shared understanding of vision or mission, shared resources and skills, mutual benefits, and good management practices [47]. In their study, Dean et al. [25] found good working relationships from previous partnerships as a contributor to effectiveness and sustainability in north-south partnerships. Going into PROJECT-2, the participants incorporated many of the experiences and lessons learned from PROJECT-1 into establishing PROJECT-2, including some of the characteristics highlighted by Buse and Tanaka [47] and Dean et al. [25], it seemed the partners understood and recognized their strengths and weaknesses going into PROJECT-2 and planned strategies to improve the functioning of the partnership.

### Mission for sustainability – a house divided cannot stand

The establishment of a clear mission and vision for partnership is important not only for role and resource distribution but also has an impact on the sustainability of projects even long after funding has ceased. An understanding of the purpose of coming together into partnership with end goals clearly understood by all partners involved is key. This includes the alignment of project mission to that of institution for sustainability. John, Ayodo, and Musoke [21] also included the same moral values as an important characteristic to effective global partnerships, this promotes trust among the partners. Even though the partners understood the importance of establishing a vision and mission for the effectiveness of the partnership prior to establishing PROJECT-2, during the interviews the participants struggled to articulate a collective understanding of the vision and mission of the partnership. The participants were pulling apart different aspects of the project objectives without a clear understanding of the overall partnership aim. However, what was interesting in their definitions of partnership mission was how the partners were linking the mission to the outputs of the project, but what was missing was the partners’ understanding of project outcomes and linking those to the institution needs and mission. Using the Theory of Change (ToC) concepts to distinguish outputs and outcomes, mission connects to overall goals linked to context and is future-orientated, whereas outputs connect to shorter term goals that contribute to fulfilment of outcomes in partnership [48, 49]. In the interviews, only one participant (Participant 10, IP) gave a definition of the mission that focused outcomes rather than outputs, moving beyond institution and partnership but also national level objectives. A clear understanding of project outcomes has an impact on sustainability which in turn influences mission and functioning of partnerships. In PROJECT-2 the partners stated that they were intentional about developing a partnership that is driven by the needs of southern partners. Working closely with stakeholders at the country level in developing a research agenda focused on nutrition priorities was a strategy implemented by the partners in ensuring sustainability of project. This also increases the chances of project impact and sustainability in the country; and long-standing challenges of global north and south partnerships [50, 51].

### Communication breakdown impacts transparency

Effective communication among the partners not only has an impact on partnership functioning but can also promote transparency in the partnership. Good work relationships established from the PROJECT-1 project were perceived as a strength to establish PROJECT-2. However, it is interesting that these good work relationships were mentioned only by the supporting partners. These perceived good work relationships should be questioned for their genuineness and relevance in PROJECT-2. The communication and working together between the partners in PROJECT-1 are seen as not transparent. When the partners in S2 institution experienced language barriers and translation issues within the institution, they did not communicate with the partners about changes implemented at the institutional level. This then undermined the view of good work relationships and brings to question what could have led S2 to implement changes in the partnership without informing the rest of the partners and funders. During the in-person meeting, an explanation was asked for and given for the changes that were implemented in S2 but the issue was not followed up post the in-person meeting to make relevant changes in the functioning of the program in S2 institution. Transparency as highlighted by Monetta et al. [26] and Nakanjako et al. [18] goes beyond financial transparency but also to challenges experienced at an institution level and the openness of partners to welcome contextual knowledge to improve partnership functioning. This means going to the root of issues at the institute level and finding contextually appropriate solutions without following stringent partnership legacies that are not beneficial to all partners.

The power of language in communication between partners is equally important in promoting respect, knowledge value, fairness, and transparency in north-south partnerships. In the interviews, one participant referred to one of the southern institutions as a “little brother” in the partnership, alluding to the partner’s contribution and distribution of resources. Using such language in north-south partnerships is paternalistic and continues the legacy of colonialism [52] and diminishes the value and knowledge value of southern partners. This could be argued to explain the reasons the southern partners did not tell the partners about changes at institution level, tied to value of partners in the partnership and knowledge value.

### Unclear role distribution and resource contributions give rise to power dynamics

Role distributions and resource contributions in partnerships can be a birthplace for inequitable power dynamics if partners do not understand the inequalities that exist within them in the partnership. These can often lead to frustrations and unmet expectations – antagony in partnerships. The changes in administrative duties and communication with funders implemented in PROJECT-2 raised issues of power dynamics within the partnership functioning. This is nothing new to global health partnerships. Historically, issues of power dynamics and equality have been and continue to be an ongoing challenge of partnerships with countries in the global north and south [32, 36, 53]; with updates in literature calling for a shift from equality *(sameness)* to equity *(fairness)* in global north-south partnerships [33]. Although many partners in PROJECT-2 believed that there was equality in the partnership, there were concerns expressed by some participants about control and power. These were implied in control and the overall decision-making power of PROJECT-2 held by the northern partners. Even then, with these concerns of power dynamics expressed by some participants, the partners did not have any effective strategies to flatten the power dynamics. Such dynamics further raises questions on collaborations with local expertise in these partnerships, the people who have better understanding of context and who are able to make decisions aligning with true needs of southern partners.

Even though there has been a shift over the years, with developments of approaches and models for engagements in north-south partnerships, issues of inequitable power dynamics and control persist in these partnerships. These are often rooted in who has control over decision-making. In as much as the PROJECT-2 partnership approached the development of the partnership from the model that puts Southern partners at the forefront of decisions about the research agenda, the partnership was at risk of falling victim to many pitfalls of global health partnerships due to overall decision-making power held by Northern partner. There have been many approaches and models developed to improve global health partnerships over the years [54, 55], in practice, many of these partnerships still struggle with challenges of power dynamics and these are often rooted in the mismatch in research priorities and research context, unclear role distributions, resources, communication, funding, and a lack of clear understanding of the research agenda [15, 54, 56, 57]. All of these could be summed up as ‘control’ and can be attributed to the power of decision-making in many of these partnerships.

Control is further highlighted in the partnership by how the partners understand each other’s resource contributions and benefits in the project. In defining contributions and benefits, the partners created a divide in the partnership by calling themselves implementing and supporting partners. Using these labels created hierarchy in the partnership with the supporting seen as experts and the implementing seen as beneficiaries of the partnership; potentially exacerbating ‘the little brother effect’ attached to global north-south partnerships [33, 52]. However, the focus should be shifted to understanding what are the hidden or unacknowledged benefits of the partners who are considered experts in partnerships between countries in the global north and south. In their study, Syed et al. [58] found that benefits for HICs in these partnerships included deeper contextual understanding of working in LMICs for future research and transferring research learnings and innovations to their countries. Dean et al. [25] further state that having a clear understanding of benefits for all partners is important, this has an impact on the effectiveness and sustainability of partnerships.

In PROJECT-2, the supporting partners see their main role as providing support in the partnership with minimal benefits directly linked to the partnership. This is an interesting perspective from the supporting partners whereas they view the partnership as an equal partnership. By definition, an equal partnership connotes that all partners contribute and benefit equally [59]. According to Crane [1] and Geissler [27] partnerships steered towards ‘global health’ should not be considered ‘partnerships’ or ‘collaborations’ because of their intrinsic nature of inequality. As stated above, literature has seen a shift in global health partnerships towards equity (fairness) instead of equality (sameness) emphasizing differences in contributions and benefits of partners [33, 60, 61]; these are fixed according to the needs of the partners.

## Study limitations

Firstly, the PROJECT-2 partnership has a small membership, so maintaining anonymity and confidentiality in the data was difficult as participants knew each other very well. Anonymity also made it difficult in presenting the research findings in this paper, we could not contextualize the quotes and certain quotes had to be removed from presentation of findings to preserve anonymity and confidentiality of participants. Secondly, the researcher (SL) was well acquainted with the research participants as she worked on the PROJECT-1 and works on PROJECT-2 as a researcher. This could have caused response bias from the participants. Thirdly, language was a barrier to communicating with one participant. For this particular participant, an interview guide was sent to answer the questions. Lastly, the use of digital platforms to conduct interviews was a challenge, the internet connection was a problem at times, and getting participants available was a challenge at times.

## Conclusion

Using the Bergen Model of Collaborative Functioning (BMCF), the study explored the development and functioning of a northern and multi-south partnership in global health. Even though the study was conducted during the initial stages of partnership development, the partners seemed to be aware of some of the underlying issues in the partnership and their potential to influence functioning. Roles and structures were experienced by the partners as possibly the main contributor to complex power dynamics. Tied to roles and structure are financial resources, partner resources, and leadership which also had an impact on distribution of roles. Lessons from the previous partnership included lack of agreement on mission and vision for the partnership, and poor communication with students, among partners, and with institutions. Even though the partners had an intention of developing vision and mission and communication strategies, these seemed to be ineffective as participants did not have a common mission and vision and the partners still maintained functioning of partnership and teaching and learning to be in English without effective solutions to mitigate those issues. A key feature usually missing in global north-south health partnerships is positioning projects based on southern needs, not only on paper but actually finding research priorities that are rooted in context and allowing southern partners to lead projects as members with the most contextual understanding. Such changes in the functioning of global health partnerships would mitigate and solidify the shift from equality to equity, therefore promoting sustainability of these projects even after funding ceases.

## Data Availability

No datasets were generated or analysed during the current study.

## Footnotes

1 All country names and institutions have been concealed in the paper to maintain anonymity and confidentiality of the partnership.

2 IP = Implementing Partner. SP = Supporting Partner.

## Abbreviations

BMCF: Bergen Model of Collaborative Functioning
HEIs: Higher Education Institutions
HICs: High Income Countries
IDIs: In–depth Interviews
LMCIs: Low–and–middle income countries
PI: Principal Investigator
ToC: Theory of Change
